# Correlation analysis and comprehensive evaluation of dam safety monitoring at Silin hydropower station

**DOI:** 10.1038/s41598-025-15094-6

**Published:** 2025-08-13

**Authors:** Weixing Yang, Tingting Li, Bo Wen, Zhixuan Miao

**Affiliations:** 1 POWERCHINA Northwest Engineering Corporation Limited, Xi’an, 710100 China; 2 Experimental Research Department, Technical Centre, Northwest Survey and Design Research Institute of China Electric Construction Group Co, Xi’an, China; 3 Safety Intelligent Monitoring Innovation Workshop, Xi’an, China; 4 Xi’an Children’s Hospital, Xi’an, 710003 China

**Keywords:** Cluster heatmap, Correlation analysis, Principal component analysis, Comprehensive evaluation, Dam safety monitoring, Environmental sciences, Hydrology, Engineering

## Abstract

Dam failures pose catastrophic risks to human life and property, necessitating robust safety monitoring systems for risk mitigation. However, the specific contributions of distinct monitoring modalities to dam safety remain inadequately characterized, particularly regarding their differential impacts on structural integrity assessment. This study investigates the correlation between diverse monitoring modalities and dam structural safety through a comprehensive analysis of the Silin Hydropower Station dam. We analyzed 324 datasets collected from nine types of monitoring sensors installed across 36 dam cross-sections. Statistical analyses including one-way ANOVA, cluster analysis, and principal component analysis (PCA) were employed to quantify the influence patterns of monitoring parameters. The safety impact levels of all 36 cross-sections were systematically ranked, establishing a prioritized reference framework to inform decision-making in dam safety management. Unlike conventional dam safety assessments that predominantly rely on subjective empirical judgments, this study introduces an objective methodology integrating principal component analysis (PCA) of heterogeneous monitoring data across multiple dam cross-sections. The analytical outcomes were systematically quantified, hierarchically ranked, and visualized through multidimensional mapping techniques. The results demonstrated that variations in fissure (X2), horizontal displacement (X3), tilt (X4), stress (X6), soil-displacement (X8), and denotes water-level (X9) exerted highly significant effects on dam safety (*p* < 0.001). The first two principal components cumulatively accounted for 74.1876% of the total variance, with eigenvalues reaching 6.6769. In the comprehensive evaluation, cross-section T4 (T4) obtained the maximum score (0.8500), while cross-section T35 (T35) showed the minimum score (0.0175). In conclusion, the analysis revealed that X9, X8, X2, X3, and X4 exerted significant impacts on dam safety, while cross-section T4 achieved the highest comprehensive evaluation score. This approach employs Principal Component Analysis (PCA) with integrated scoring to reduce multivariate dimensionality, enabling rapid identification of key monitoring sections critical to dam safety, and demonstrates broad applicability for dam safety monitoring.

## Introduction

Dam breaches can trigger catastrophic consequences, including extensive loss of life and irreversible damage to downstream infrastructure, ecosystems, and socioeconomic systems^1–3^. Dam failure is typically the result of the combined effects of multiple factors, and the significance of different monitoring indicators varies depending on the type of dam, materials used, and environmental conditions. According to international research and engineering reports (such as those from ICOLD, USBR, and ASDSO), the approximate proportions of the nine categories of monitoring parameters in dam failure causes are as follows: seepage and internal erosion account for about 30–40% (dominant factors), cracks account for about 15–25%, abnormal water-level accounts for about 20–30% (often a triggering factor), the combined proportion of sedimentation and horizontal displacement accounts for about 10–20%, stress and load account for about 10–15%, soil-displacement accounts for about 5–10%, and tilt independently accounts for less than 5% (usually coupled with other indicators)^4,5^.

The cascading impacts of such events often defy precise quantification due to their nonlinear interdependencies and long-term cascading effects^6,7^. The 2018 catastrophic failure of a subsidiary dam at the Xe-Pian Xe-Namnoy hydropower project in Laos stranded approximately 13,000 individuals and displaced over 6000 residents due to rapid-onset flooding (World Bank, 2019). In 2021, the Edenville Dam failure in Michigan, USA, triggered by extreme precipitation events, propagated downstream breaches at the Sanford Dam, necessitating mass evacuations (ASCE, 2022). A 2023 storm-induced collapse of the Derna Dam in Libya resulted in catastrophic flooding, with fatalities estimated at 11,000 according to United Nations reports. Most recently, on April 5, 2024, a dam breach in Orsk City, Orenburg Oblast, Russia, inundated 10,300 residential structures and 13,100 land parcels, prompting the evacuation of 6,555 individuals, including 1597 children (local government preliminary data)^3,8^. These cases represent a non-exhaustive sample of documented dam failure incidents, with analogous events recurrently reported across diverse geographical and climatic contexts (ICOLD Disaster Database, 2023; EM-DAT, 2024). The etiology of dam breaches is multifactorial^9^, involving interacting drivers such as natural hazards, operational mismanagement^10^, technological inadequacies, and deficiencies in predictive capabilities^11–13^. Current mitigation strategies predominantly focus on meteorological forecasting enhancements^14^, rigorous dam maintenance protocols^15^, and preemptive structural reinforcement initiatives^16^. However, empirical evidence from recent failures (e.g., 2023 Derna Dam collapse; UN OCHA, 2023) underscores persistent residual risks attributable to system interdependencies and escalating hydroclimatic extremes that exceed conventional engineering thresholds^11,17,18^.

Traditional monitoring methods predominantly rely on empirical analysis, which are characterized by strong subjective judgment and fail to comprehensively evaluate the integrated effects of multiple monitoring cross-sections on dam safety. For instance: Fang et al.^9^ investigated the impact of concrete dam deformation (an external monitoring indicator) on structural integrity, while Chang et al. assessed the relationship between internal monitoring parameters (e.g., anchor rod stress) and slope stability. Although these studies have independently demonstrated the influences of individual monitoring parameters on dam behavior, they lack systematic integration to evaluate the comprehensive interactions between multiple monitoring cross-sections and overall dam safety^19,20^. Specifically, critical parameters such as external deformation and internal stress are analyzed in isolation without establishing quantitative correlations between external and internal monitoring systems^21,22^.

Despite the extensive deployment of embedded sensors within and around dam structures for real-time safety monitoring, current systems remain unable to reliably predict the temporal occurrence and primary triggers of dam breaches^21,22^. This persistent uncertainty underscores unresolved mechanistic interdependencies underlying breach causality, necessitating advanced interdisciplinary investigations integrating geotechnical diagnostics with stochastic hazard modeling^23–25^. A preliminary analysis of the data collected from dam safety monitoring sensors indicates that parameters Sedimentation, Fissure, Horizontal Displacement, Tilt, Seepage, Stress, Loading and Soil-displacement exhibited noticeable variations prior to dam failure^26–28^. However, the inter-correlation degrees among different dam cross-sections and monitoring types regarding dam safety, along with the effectiveness of principal component analysis (PCA) and comprehensive evaluation methods for assessing these correlations and impacts, have not been conclusively established through systematic validation^6,9,29,30^.

To address this research gap, the present study establishes a systematic framework focusing on the Silin Hydropower Station dam. We deployed a continuous monitoring system comprising nine distinct sensor types, strategically distributed across both internal and external dam structures. The acquired safety monitoring datasets underwent rigorous multivariate analysis, including: (1) correlation coefficient evaluation^31,32^; (2) hierarchical cluster analysis; (3) principal component analysis (PCA); and (4) development of a composite evaluation index system. This study aims to (1) quantify contribution rates and eigenvalues of principal components governing dam safety, (2) decipher inter-correlation patterns within heterogeneous monitoring datasets, and (3) establish cross-sectional evaluation metrics through an integrated analytical framework implemented for comprehensive dam assessment^9,33,34^.

## Materials and methods

### Study area

**Fig. 1:**
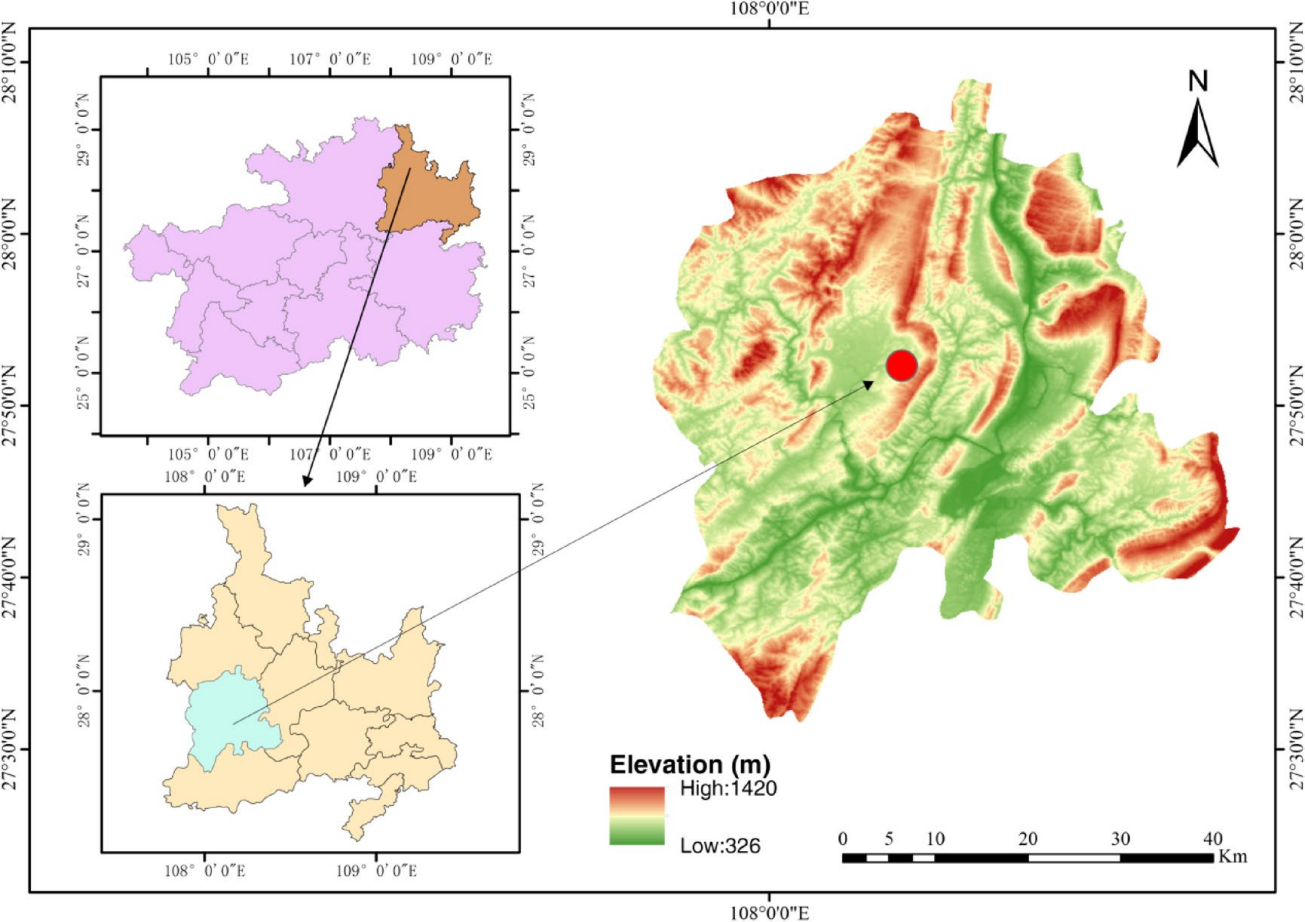
Digital elevation map of the location of Silin Hydropower Station. The figure comprises three panels: Left upper panel: Administrative map of Guizhou Province, Southwest China. Left lower panel: Detailed topographic map of Tongren City. Right panel: High-resolution (30 m) Digital Elevation Model (DEM) of Sinan County, with a solid red circle (5 km buffer radius) marking the geolocation of Siling Hydropower Station. Figure 1 was originally created by myself using the open-source software QGIS (version 3.40.7, URL: https://www.gnu.org).

For this study, all monitoring data were obtained from the monitoring system of the Si Lin hydroelectric dam. Silin Hydropower Station is located on the Wujiang River in Sinan County (107° 52′–108° 28′ E, 27° 32′–28° 10′ N), Tongren City, Guizhou Province (Fig. 1). It is a key provincial project in Guizhou and a backbone facility of the ‘West–East Electricity Transmission’ initiative. As part of the Wujiang River Cascade Development Plan, it serves as the sixth-tier hydropower station on the main stream of the river. The Silin Hydropower Station features a roller-compacted concrete gravity dam with a crest elevation of 452 m. It has a maximum dam height of 117 m and a total crest length of 326.5 m. With a total installed capacity of 1,050 MW, the power station primarily serves electricity generation purposes, followed by navigation functions, while also incorporating flood control, irrigation, and other comprehensive benefits. The region has a maximum altitude of 1,420 m and a minimum altitude of 326 m, with an average temperature of 17.3 °C (Fig. 1). January is the coldest month and July is the hottest. The region has recorded extreme temperatures, reaching 40.7 °C and dropping to − 5.5 °C.

## Experimental design

To facilitate readability and comprehension, the nine types of safety monitoring in this study are denoted as follows: X1 to X9 represent sedimentation, fissure, horizontal displacement, tilt, seepage, stress, loading, soil-displacement, and water-level, respectively.

**Fig. 2:**
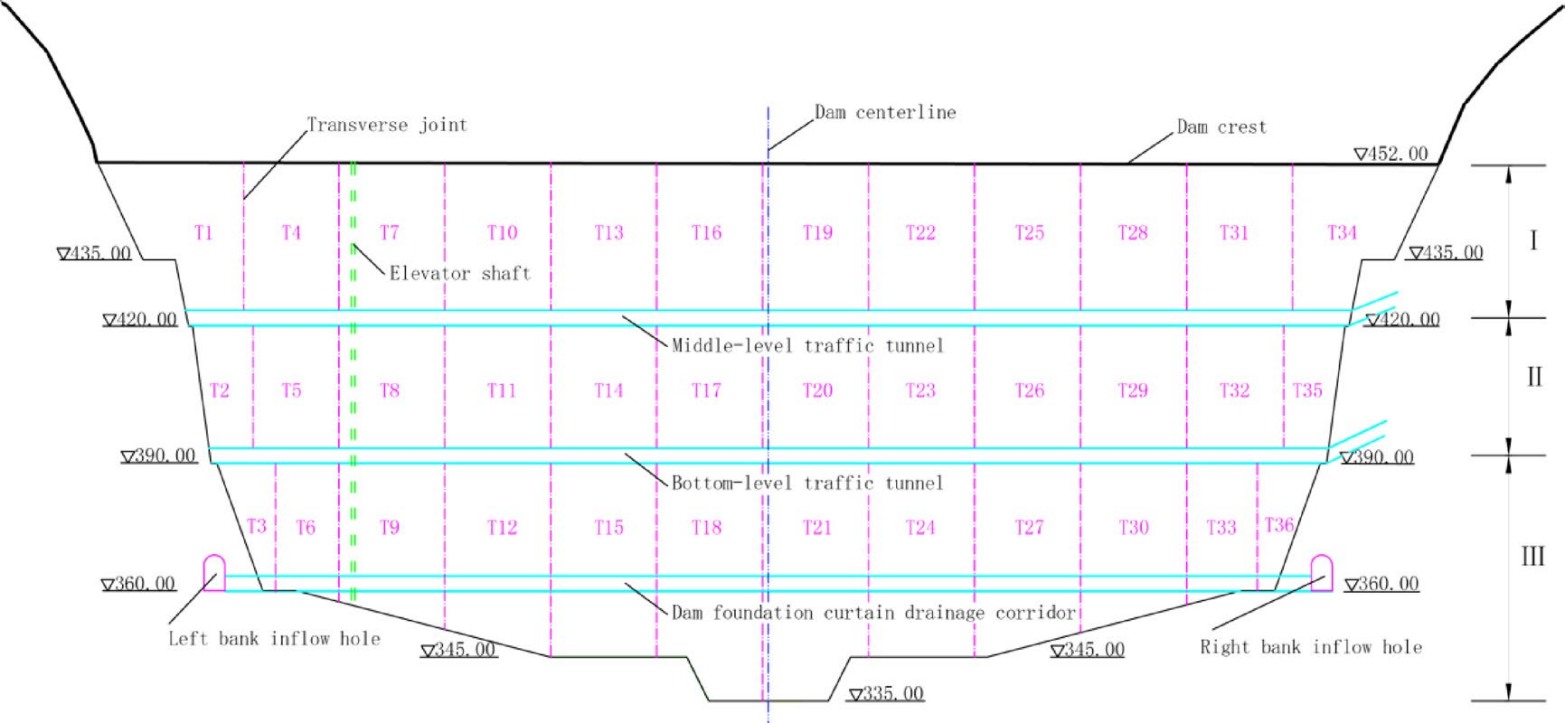
Schematic Layout of Monitoring Cross-Sections at Silin Hydropower Station Dam. The magenta dashed line in the figure represents the ‘Transverse joint’. The area between the two green dashed lines is the ‘Elevator shaft’. The blue dashed line indicates the ‘Dam centerline’. The horizontal thick black solid line is the ‘Dam crest’. The black solid lines on the left and right sides represent the mountain bodies on both sides of the dam. The letters I, II and III show the dam monitoring areas. The inverted triangles are elevation symbols. The cyan solid—line areas sequentially indicate the 'Middle—level traffic tunnel’, 'Bottom—level traffic tunnel’ and 'Dam foundation curtain drainage corridor’. The magenta solid—line horseshoe shape represents the 'bank inflow hole’. Magenta alphanumeric codes (e.g., T1) denote monitoring cross-section identifiers, where the letter T represents the transverse axis and the numeric suffix indicates sequential numbering (No. 1–36).

For correlative analysis and comprehensive evaluation of dam safety monitoring data at this hydropower station, the dam has been divided into 36 monitoring cross-sections based on structural transverse joints. These cross-sections are distributed across three dam areas, with each area containing 12 monitoring cross-sections. Each section has been equipped with nine types of sensors for structural safety monitoring, resulting in the acquisition of 324 distinct monitoring data points. The collected monitoring data has been averaged across multiple measurements within the same time period as the monitoring site’s final dataset, and we have performed preliminary classification and organization of the processed data (Table 1 and Fig. 2).

**Table 1:** Distribution of the data.

Cross-section	Area	Sensor category
T1 T4 T7 T10 T13 T16 T19 T22 T25 T28 T31 T34	I	Sedimentation Fissure Horizontal Displacement Tilt Seepage Stress Loading Soil-displacement Water-level
T2 T5 T8 T11 T14 T17 T20 T23 T26 T29 T32 T35	II	
T3 T6 T9 T12 T15 T18 T21 T24 T27 T30 T33 T36	III

To ensure the accuracy of analytical results, data collection at each monitoring site was conducted through integrated automated and manual protocols. A minimum of three independent measurements were obtained from each monitoring site, with the final result determined by averaging these measurements. The acquired data underwent preliminary quality assessment to verify compliance with sample size requirements and evaluate distribution patterns of target analytes.

Concurrently, the monitoring data were classified as internal (X1, X2 and X3) and external (X4, X5, X6, X7, X8 and X9) (based on their sources). Second, to analyze the quality of the monitoring data, we visualized the distribution and dispersion by plotting box plots with normal distribution reference lines^9,35^.

Finally, we conducted the Kaiser–Meyer–Olkin (KMO) test and Bartlett’s test of sphericity to ensure that the KMO value exceeded 0.6 and the *p*-value of Bartlett’s test was less than 0.05. We conducted the Kaiser–Meyer–Olkin (KMO) test and Bartlett’s test of sphericity to ensure that the KMO value exceeded 0.6 and the *p*-value of Bartlett’s test was less than 0.05. Statistical analyses were performed using IBM SPSS Statistics 26 and complementary software packages. Initial assessments included normality tests (Shapiro–Wilk/Kolmogorov–Smirnov) and homogeneity of variance evaluation (Levene’s test). The Kaiser–Meyer–Olkin (KMO) measure and Bartlett’s sphericity test were subsequently conducted, with predetermined thresholds of KMO > 0.6 and Bartlett’s *p*-value < 0.05. Multivariate analyses encompassed Pearson/Spearman correlation matrices, hierarchical clustering with and principal component analysis (PCA) employing varimax rotation. Analytical outcomes were visualized through OriginPro 2024 and R-4.4.2 generated heatmaps, dendrograms, and PCA biplots.

To test the data sensitivity of nine types of sensors in dam safety monitoring, 324 datasets from 36 monitoring sections were min–max normalised under the same environmental conditions. The integrated sensitivity indices of the nine sensor types for each section were then calculated^36,37^.

To ensure comparability across different measurement scales, the raw monitoring data were normalized using the Z-score min–max normalization method. The calculation formula is as follows:

1 $$Z=\frac{X-{X}_{min}}{{X}_{max}-{X}_{min}}$$

where $Z$ represents the normalized value, $X$ denotes the original value, and ${X}_{max}$ and ${X}_{min}$ indicate the maximum and minimum values in the monitoring dataset of the corresponding type, respectively.

In dam safety monitoring, to further explore the potential relationship between the 36 dam sections and the 9 types of sensors and to provide a scientific basis for decision-makers, we analyzed the correlation in the monitoring data and marked the significant differences among the data monitored by the 9 sensor types using the alphabetical method.

To reveal the intrinsic structure and detect anomalies in dam safety monitoring data, 36 sections and 9 categories of monitoring data were analyzed using cluster analysis and visualized through a cluster heat map. In addition, to further explore the extent to which the 36 faults (of the 9 monitored types) affect dam safety, we conducted a membership function analysis.

Principal component analysis was performed on the dam monitoring data with as little loss of information as possible. The main processes of principal component analysis include standardisation of raw data, calculation of a covariance matrix or correlation coefficient matrix, eigenvalue decomposition, selection of the number of principal components, and generation of principal component scores.

### Statistical analysis

The initial collation and analysis of dam safety monitoring data were conducted using Microsoft Excel 2016. The digital elevation model (DEM) map was generated by processing the data in QGIS (version 3.40.7, https://www.gnu.org). Correlation analysis and principal component analysis of dam safety monitoring data were performed using IBM SPSS Statistics 26 (SPSS Inc., USA). One-way ANOVA and Pearson correlation analysis were employed to assess the significance (*p* < 0.05) and correlation of the data. The results of the analyses were visualized using OriginPro 2024 (OriginLab Corporation, USA). Meanwhile, we used R environment (version: R-4.4.2, https://www.r-project.org/) to draw correlation heatmaps.

## Results

### Data quality analysis of various types of observations

A box plot is a statistical chart used to depict the dispersion of a dataset. It can reflect the stability of optimization effectiveness. In box plots, the dispersion of data is measured by the interquartile range (IQR).

**Fig. 3:**
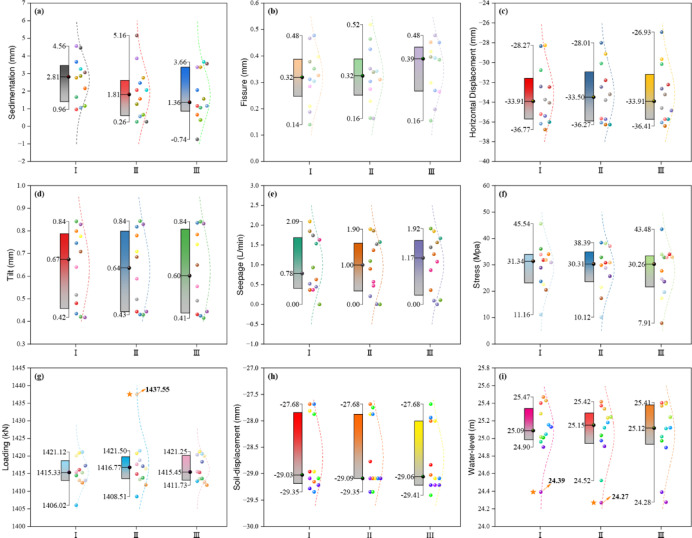
Box diagrams of the 9 monitoring types located in different areas. (**a**)–(**i**) sequentially display box plots of monitoring data distributions for: sedimentation, fissure, horizontal displacement, tilt, seepage, stress, loading, soil-displacement, and water-level variations.

From Fig. 3, three data outliers were identified in the 324 sets of monitoring data. In X7, an extreme value of 1437.55 KN was observed at location II (Fig. 3g). In X9, extreme values of 24.39 m and 24.27 m were recorded at locations I and II, respectively (Fig. 3i). These results indicate that localized extreme values are prone to occur in both X7 and X9 monitoring during dam safety assessments. The data analysis of the 9 monitoring types is shown in the following Table 2 and Fig. 3.

**Table 2:** Data analysis results of 9 types of monitoring.

Type of monitoring	Smallest IQR	Area	Note
Sedimentation (mm)	2.00	II	This indicates that the data variation in this area is the smallest
Fissure (mm)	0.14	II	
Horizontal Displacement (mm)	4.11	II	
Tilt (mm)	0.33	I	
Seepage (L/min)	1.21	II	
Stress (Mpa)	10.83	I	
Loading (kN)	5.61	I	
Soil-displacement (mm)	1.21	II	
Water-level (m)	0.35	II	

Based on the analysis of the data above, it was found that only three out of the 324 data groups exhibited extreme values, while the remaining data showed lower dispersion and followed a normal distribution with random characteristics.

The degree of sensitivity is represented by the height of the lollipop column, with greater height corresponding to higher sensitivity. This suggests that the monitoring sensors in this section are exposed to significant external variations, resulting in more pronounced fluctuations in the monitored data.

As shown in Fig. 3, the highest sensitivity value (6.20) occurs in T4, while the lowest (2.24) is observed in T29. Notably, the majority of cross-section exhibit sensitivity values exceeding 3.72.

### Correlation analysis

To further investigate the presence and strength of correlations between different monitoring types across areas, we performed both one-way ANOVA and Pearson correlation analyses.

It was found that the monitoring-type data from areas I and II showed significant differences among groups X3, X6, X7, X8, and X9, whereas no significant differences were observed among groups X1, X2, X4, and X5 (Table 3). In area III, significant differences exist among monitoring types X3, X7, and X8 compared to other monitoring types. However, no significant differences are observed between types X1, X2, X4, and X5, nor between types X6 and X9 (Table 3).

**Table 3:** Description of evaluation results by monitoring type.

Monitoring type	Area		
	I	II	III
Sedimentation (mm)	2.64 ± 1.26d	1.91 ± 1.50d	1.77 ± 1.44c
Fissure (mm)	0.32 ± 0.10d	0.32 ± 0.11d	0.35 ± 0.11c
Horizontal Displacement (mm)	− 33.31 ± 2.93f	− 33.21 ± 2.95f	− 33.19 ± 2.99e
Tilt (mm)	0.63 ± 0.17d	0.62 ± 0.17d	0.62 ± 0.18c
Seepage (L/min)	1.01 ± 0.72d	0.96 ± 0.70d	1.02 ± 0.72c
Stress (MPa)	29.23 ± 8.80b	28.44 ± 8.61b	27.56 ± 9.52b
Loading (KN)	1415.43 ± 4.24a	1417.68 ± 7.27a	1416.31 ± 3.58a
Soil-displacement (mm)	− 28.68 ± 0.69e	− 28.67 ± 0.67e	− 28.73 ± 0.63d
Water-level (m)	25.1 ± 0.29c	25.05 ± 0.35c	25.05 ± 0.38b

**Fig. 4:**
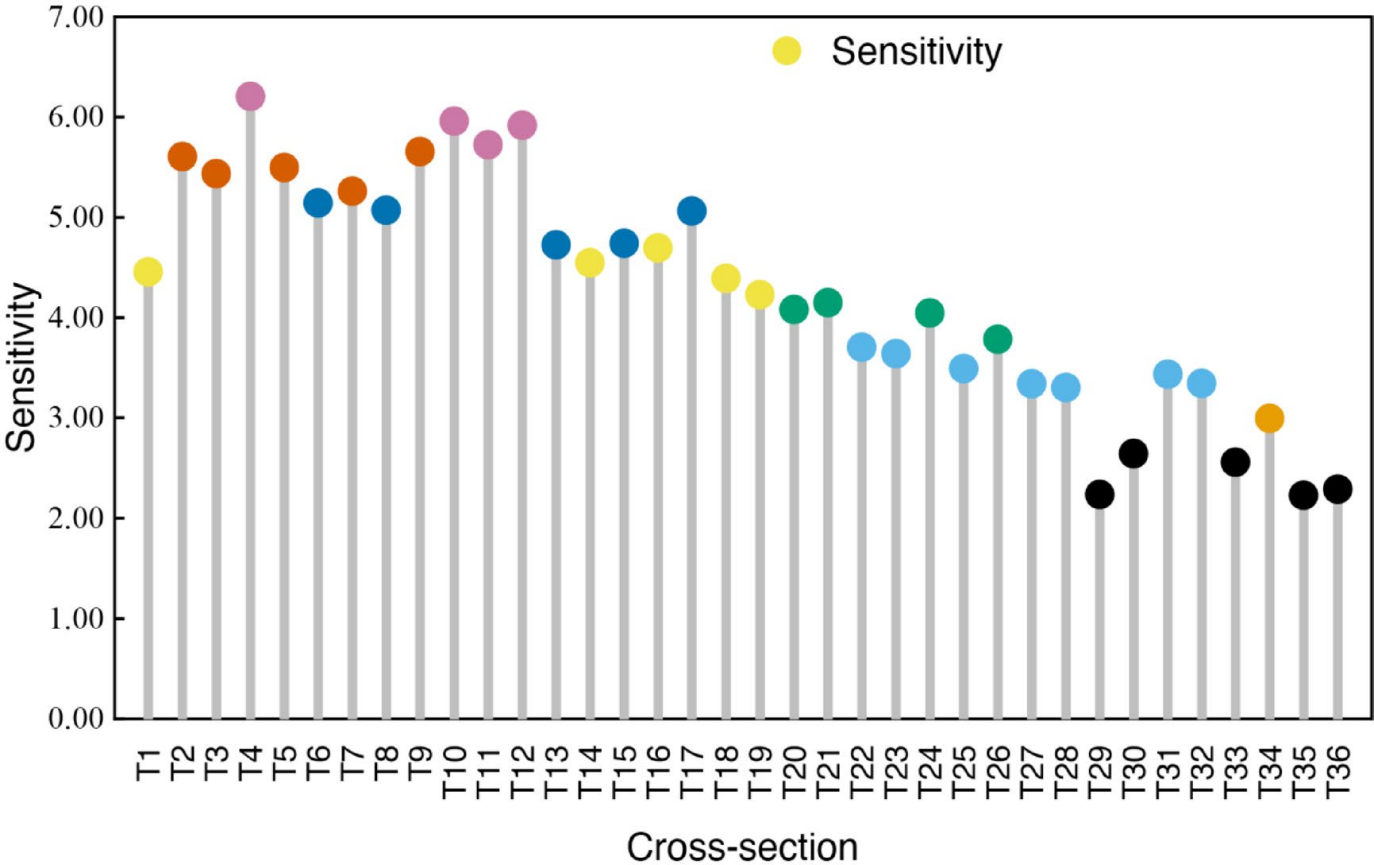
Lollipop chart of composite sensitivity values for 36 cross-section sensor monitoring data. The length of the bars in the figure indicates the magnitude of the sensitivity value for each monitoring section.

Data represent mean values ± standard error (Fig. 4). Values within a column sharing the same superscript letter are not significantly different (*p* > 0.05, one-way ANOVA).

**Fig. 5:**
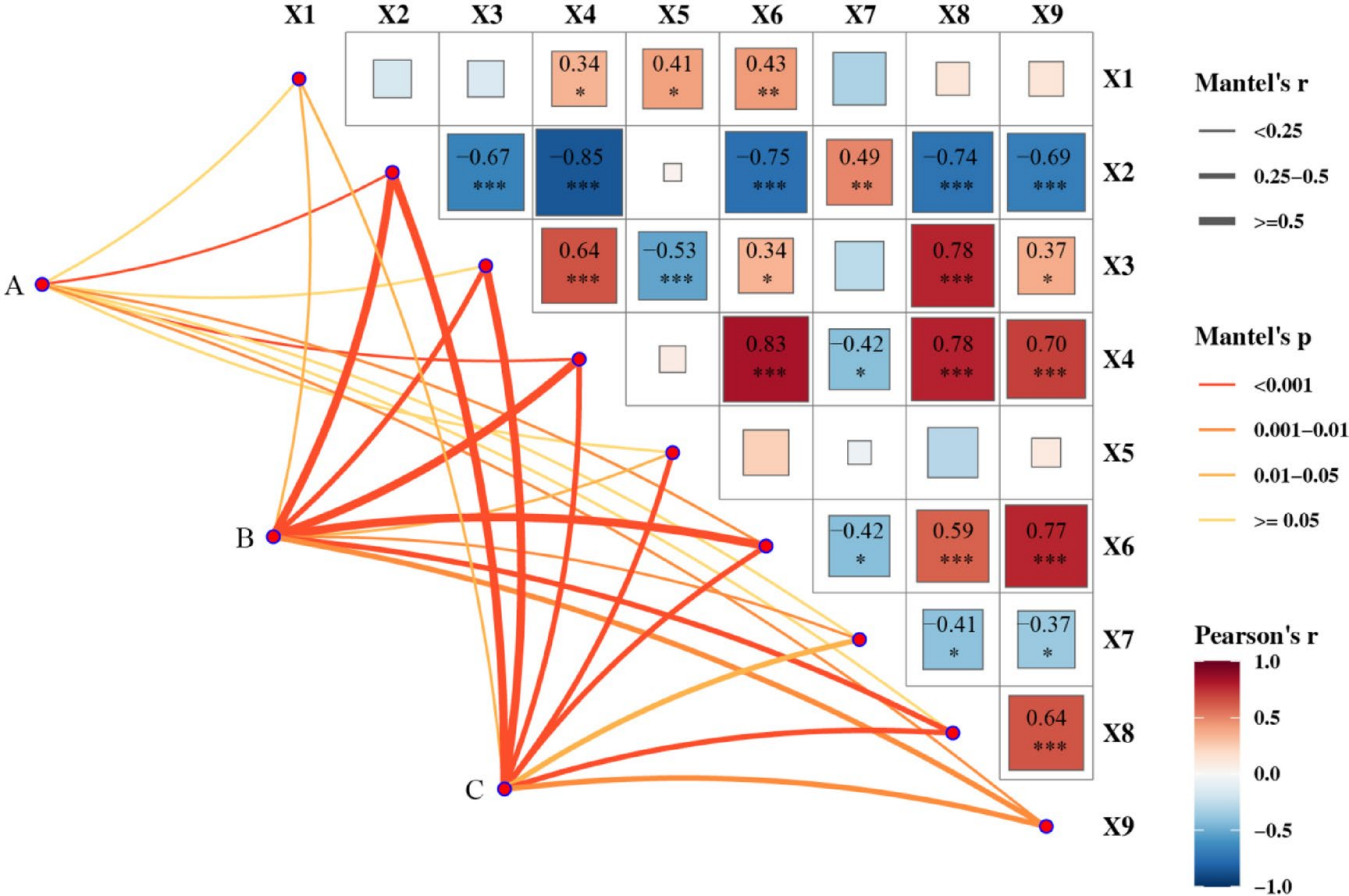
Correlation analysis between 9 types of monitoring data. The 3 dots on the left indicate the 3 subgroups. The right side indicates a heat map of the correlation between each monitoring category, with the colours indicating the correlation coefficients, *indicating significant correlation at the 0.05 level, **indicating significant correlation at the 0.01 level, and ***indicating significant correlation at the 0.001 level. The width and color of the middle connecting sections correspond to the r statistic and significant *p*-value, respectively.

Based on further analysis of Fig. 5, our study revealed significant correlations among key parameters. X1 showed significant positive correlations with X4, X5, and X6 (r = 0.34, 0.41, and 0.43, respectively). X2 exhibited negative correlations with X3, X4, X6, X8, and X9 (r = − 0.67, − 0.85, − 0.75, − 0.74, − 0.69), while demonstrating a positive correlation with load (r = 0.49). X3 was positively correlated with X4, X6, X8, and X9 (r = 0.64, 0.34, 0.78, 0.37), but negatively correlated with X5 (r =− 0.53). X4 displayed positive correlations with X6, X8, and X9 (r = 0.83, 0.78, 0.70), contrasting with its negative correlation with X7 (r =− 0.42). X6 maintained positive correlations with both soil- displacement and X9 (r = 0.59, 0.77), while showing an inverse relationship with load (r =− 0.42). X7 demonstrated negative correlations with X8 and X9 (r =− 0.41, − 0.37), whereas a positive correlation persisted between X8 and X9 (r = 0.64).

### Cluster analysis and membership function

To investigate the intrinsic factors influencing dam safety and their impact magnitudes more comprehensively, cluster analysis and membership function analysis were conducted on various monitoring types distributed across different dam sections.

**Fig. 6:**
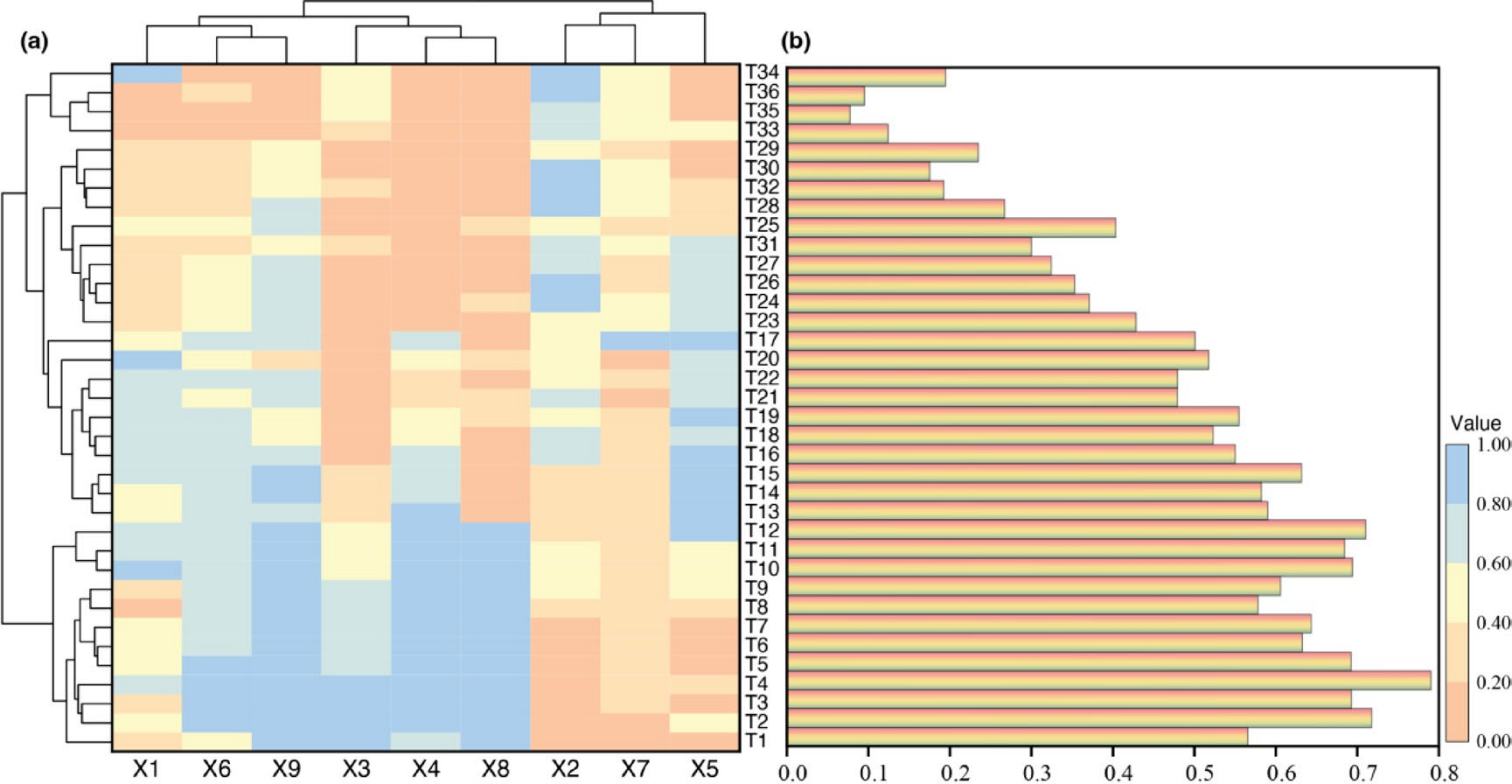
Clustered heatmaps and membership functions across-sections and monitoring types. (**a**) heatmap of cluster analysis between cross-sections and between monitoring types. (**b**) cross-sectional membership function.

The results of the study showed that the 36 sections could be divided into three groups: T34, T36, T35 and T33 as the first group with the lowest composite scores, T1-T20 as the second group with the highest composite scores, and the remaining sections forming the third group with intermediate composite scores (Fig. 6a). The monitoring dataset comprising nine categories exhibited a bipartite classification pattern, with categories X2, X7, and X5 aggregating into one distinct group, while the residual categories collectively formed the complementary group. The highest overall score was observed in T4, followed by T2, while the lowest score was recorded in T35 (Fig. 6b).

### Principal component analysis (PCA)

In order to comprehensively evaluate the extent to which the monitoring data are specific to dam safety monitoring, the high-dimensional data were transformed into a low-dimensional representation (principal component analysis) while retaining as much key information as possible from the original data.

**Fig. 7:**
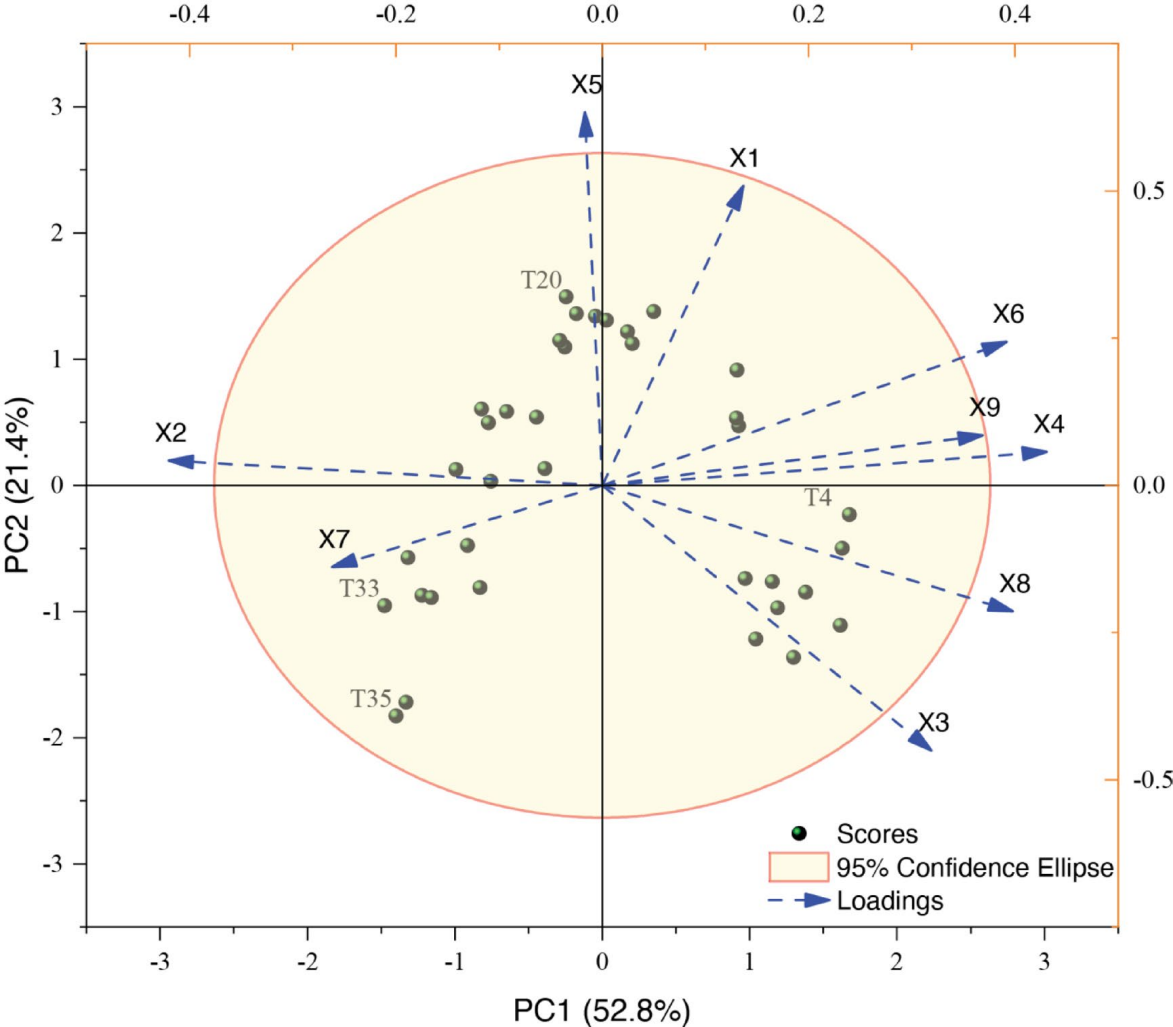
Principal component analysis of monitoring categories. PC1 & PC2: First and second principal components, with PC1 explaining 52.8% of the total variance. Green spheres: Sample scores representing individual observations in the reduced-dimensional space. Red elliptical boundary: 95% confidence ellipse for group-wise data distribution. Blue dashed arrows: Variable loadings indicating the contribution of original features to each principal component (arrow length proportional to loading magnitude).

Principal component analysis (PCA) was performed on the nine monitoring indicators. The results demonstrated that the first two principal components accounted for 74.1876% of the total variance, capturing the majority of variation information in the monitoring indicators, with a cumulative eigenvalue of 6.6769 (Table 4 and Fig. 7). The eigenvalue of PC1 is 4.7479, accounting for 52.7546% of the total variance, with the variables X4 (loading = 0.4309) and X6 (loading = 0.3922) demonstrating the most significant influence (Table 4 and Fig. 7). The eigenvalue of PC2 is 1.9290, accounting for 21.4329% of the total variance, with the variables X5 (loading = 0.6338) and X1 (loading = 0.5088) demonstrating the most significant contributions (Table 4 and Fig. 7).

**Table 4:** Principal component loadings, eigenvalues, and variance contributions.

Monitoring type	Principal component analysis	
	PC1 (52.8%)	PC2 (21.4%)
Sedimentation (mm)	0.1368	0.5088
Fissure (mm)	− 0.4203	0.0421
Horizontal Displacement (mm)	0.3193	− 0.4504
Tilt (mm)	0.4309	0.0567
Seepage (L/min)	− 0.0168	0.6338
Stress (Mpa)	0.3922	0.2435
Loading (kN)	− 0.2621	− 0.1391
Soil-displacement (mm)	0.3980	− 0.2139
Water-level (m)	0.3686	0.0850
Eigenvalues	4.7479	1.9290
Rate of variance/%	52.7546	21.4329
Rate of cumulative variances/%	52.7546	74.1876

Furthermore, this study also revealed that a comprehensive assessment of impact magnitude on dam safety monitoring demonstrated T4 exhibited the highest composite score (0.8500), followed by T2 (0.8155), while T35 showed the lowest composite score (0.0175) (Table 5).

**Table 5:** Principal component scores and composite rankings of each Cross-section.

Cross-section	Score		Composite score	Rankings	Cross-section	Score		Composite score	Rankings
	F_1_	F_2_	F			F_1_	F_2_	F	
T1	0.8802	0.1397	0.6663	11	T19	0.4768	0.9443	0.6119	16
T2	0.9843	0.3999	0.8155	2	T20	0.3905	1.0000	0.5666	19
T3	0.9809	0.2169	0.7602	4	T21	0.3877	0.8802	0.5300	20
T4	1.0000	0.4806	0.8500	1	T22	0.3771	0.8960	0.5270	21
T5	0.9059	0.2952	0.7294	7	T23	0.3268	0.7134	0.4385	22
T6	0.8456	0.2583	0.6759	10	T24	0.2086	0.7326	0.3600	26
T7	0.8345	0.3210	0.6862	9	T25	0.3451	0.5907	0.4160	23
T8	0.7989	0.1841	0.6213	15	T26	0.2230	0.6995	0.3606	25
T9	0.7758	0.3283	0.6465	12	T27	0.2285	0.5599	0.3242	27
T10	0.7616	0.6923	0.7415	6	T28	0.1780	0.4070	0.2442	29
T11	0.7572	0.7108	0.7438	5	T29	0.2051	0.3065	0.2344	30
T12	0.7582	0.8256	0.7777	3	T30	0.0800	0.2877	0.1400	33
T13	0.5227	0.9170	0.6366	13	T31	0.1534	0.5880	0.2789	28
T14	0.5328	0.8889	0.6357	14	T32	0.1007	0.2831	0.1534	31
T15	0.5792	0.9652	0.6907	8	T33	0.0000	0.2636	0.0761	34
T16	0.4538	0.9541	0.5983	17	T34	0.0501	0.3777	0.1447	32
T17	0.2621	0.7268	0.3964	24	T35	0.0246	0.0000	0.0175	36
T18	0.4128	0.9602	0.5709	18	T36	0.0460	0.0331	0.0423	35

## Discussion

Although three minor outliers were identified in this study, they accounted for only 0.93% of the total sample size (N = 324). Subsequent analyses confirmed that retaining or removing these outliers had negligible effects on the overall study outcomes. To ensure data authenticity and integrity, these values were retained in the final analysis, consistent with best practices for preserving observational accuracy in environmental monitoring studies (Fig. 3).

Our study revealed significant correlations between dam safety and monitoring indicators (categories) as well as monitoring cross-sections. Cluster analysis partitioned the nine monitoring categories into two distinct clusters, while the 36 monitoring cross-sections were categorized into three clusters. Principal component analysis demonstrated that the first two principal components accounted for over 74.2% of the total variance in dam safety monitoring data. In terms of comprehensive impact scores, T4 exhibited the highest influence on dam safety, followed by T2, with T35 demonstrating the lowest impact. Our findings demonstrated significant correlations among monitoring categories, along with hierarchical clustering patterns between monitoring types and cross-sections. It is worth noting that these results are basically consistent with those reported by Fang et al. in their research on concrete dams, and empirical data also proves this^9^.

Contrary to established methodologies in the field, our investigation unexpectedly identified isolated statistical outliers within both water level and load monitoring datasets^8,10^. While these anomalies may originate from human operational errors during manual data acquisition, robustness testing confirmed their negligible impact on the overall analytical outcomes, thereby preserving the validity of the derived conclusions (Fig. 3g and h).

Further analysis revealed that in dam safety monitoring, section T4 exhibited the highest sensitivity index, while section T35 displayed the lowest sensitivity index. This finding aligns consistently with the results from the comprehensive safety assessment of the dam Sects.^30,37^. This concordance demonstrates a strong correlation between structural integrity and the composite sensitivity scores of monitoring cross-sections (Figs. 4, 6b, Table 5), thereby validating the methodological coherence of the evaluation framework.

In this study, monitoring data from area I and II exhibited significant differences in X3, X6, X7, X8, and X9, whereas no notable variations were observed in X1, X2, X4, and X5 (Table 3). In area III, X3, X7, and X8 showed significant divergences compared to other monitoring parameters. However, no statistically significant differences were detected among X1, X2, X4, and X5, nor between X6 and X9 (Table 3). The most probable cause of this phenomenon lies in the distinct geotechnical properties, external X7 types (lateral versus vertical), and hydrogeological conditions across area I, II, and III, which led to divergent responses in X3, X6, X8, and X9. In contrast, parameters such as X1 and X2 likely remained statistically indistinguishable due to homogeneous design practices, long-term stabilization, or limitations in monitoring sensitivity.

Notably, our investigation revealed an unexpected clustering pattern: while X1, X2, and X3 were theoretically anticipated to form one distinct cluster, with X4, X5, X6, X7, X8, and X9 comprising a separate category, the empirical cluster analysis demonstrated divergent grouping. Specifically, X2, X7, and X5 coalesced into one cluster, whereas X1, X3, X4, X6, X8, and X9 formed another distinct group^38,39^. This discrepancy between theoretical expectations and empirical clustering outcomes may be attributed to inherent heterogeneity within the dataset, potentially reflecting complex interactions among underlying variables that conventional classification frameworks failed to capture (Fig. 6a).

Furthermore, significant inter-correlations were identified among the nine sensor categories, highlighting their essential functional interdependence within dam safety monitoring systems^27,40,41^. Statistical analysis revealed highly significant correlations (*p* < 0.001) among key monitoring parameters: X9 demonstrated robust associations with X2, X4, X6, and X8, while X8 exhibited strong interconnections with X2, X3, X4, and X6. Additionally, X2 showed significant correlations with X3, X4, and X6. The strong correlation primarily arises from ground X9 fluctuations altering the effective stress state in geological formations. In saturated soil layers, rising X9 increase pore water pressure, which reduces effective X6 according to Terzaghi’s principle of effective stress. Conversely, declining X9 decrease pore water pressure, thereby enhancing effective X6. These variations in effective stress subsequently modify the mechanical properties and equilibrium of soil/rock masses, leading to abrupt stress redistributions. Ultimately, such mechanical perturbations induce X8, resulting in dam X4 and X2 propagation. These findings indicate a hierarchical pattern of influence on dam safety parameters, with X9 demonstrating the strongest association, followed sequentially by X8 and X2. The consistent statistical significance (*p* < 0.001) across these relationships underscores their critical importance in dam safety monitoring systems, highlighting the predominant influence of X9 in the parameter network (Fig. 5).

Moreover, this study employs dimensionality reduction techniques to simplify multiple monitoring indicators into a few composite indices while preserving maximal original data information, aiming to reveal the underlying factors contributing to variations among monitoring types^8,29,42^. The first two principal components cumulatively accounted for over 74.2% of the total variance, sufficiently representing the majority of dam safety monitoring information while objectively revealing the intrinsic correlations among monitoring parameters. Notably, monitoring variables X2 and X4 demonstrated the highest contribution rates, a result that substantiates the initial hypotheses proposed in prior studies (Table 4 and Fig. 7). The composite membership function scores quantitatively characterize the comprehensive safety impact characteristics of monitoring cross-sections on dam structural integrity. Our analytical framework revealed significant variations in evaluation outcomes, with T4 demonstrating the highest score (0.8500), followed by T2 (0.8155), while T35 exhibited the minimum value (0.0175). This quantification establishes that T4 exerts the most significant influence in dam safety monitoring evaluations, followed sequentially by T2, with T35 showing negligible impact within the current structural safety evaluation framework (Table 5).

T4 and T2 are on the left side of the Silin Dam, near the rock—dominated hillside, with less artificial disturbance. So T4 has the highest safety score in the integrated safety monitoring evaluation, and T2 is next. Section T35 is on the right side of the dam, near the hillside. It’s between the Middle-level traffic tunnel and Bottom-level traffic tunnel portals, so it’s easily affected by external factors. Thus, its safety score is the lowest (Fig. 2). Since T4 scored the highest in the comprehensive safety evaluation, indicating the strongest stability, it is suggested that safety monitoring frequency can be appropriately reduced. In contrast, T35 scored the lowest, implying the need for increased monitoring frequency. Recommendation: power station managers should focus closely on the hillside near the T35 monitoring section, and reinforce it with local grouting if necessary to prevent disasters and address potential risks beforehand.

Our study aligns with the internal monitoring implications for safety assessment highlighted in prior research by Chang et al. on the durability failure risks of anchor bars under long-term hydro-thermal conditions in slope instability scenarios^22^, while also relating to the external monitoring impacts on dam safety demonstrated in experimental studies by Liu et al.^16^. Regarding the breaching processes and characteristics of landslide dams under varying inflow conditions. Distinctively, our work integrates both internal and external monitoring approaches to establish a comprehensive evaluation framework for dam safety monitoring. This dual-perspective methodology advances existing research by more holistically revealing the underlying factors influencing critical dam risks compared to isolated monitoring strategies reported in earlier literature.

One limitation of this study lies in the potential influence of varying time spans and sensor coverage ranges across multiple monitoring data types on the results. To address this, our team proposes that future research should focus on refining models by integrating long-term monitoring data and conducting comparative multi-case studies.

Building upon this empirical foundation, our longitudinal research program will employ a methodological framework to systematically examine the cross-variable interdependencies among X1, X2, X3, X4, X5, X6, X7, X8 and X9 monitoring parameters, with particular focus on their cumulative impacts on dam structural performance. This sustained safety surveillance initiative aims to establish temporal causality patterns while providing empirical validation for the derived relationships through continuous multi-parameter monitoring.

## Conclusion

This study conducted a comprehensive safety evaluation of dams through correlation analysis, cluster analysis, principal component analysis, and membership function analysis to explore the primary factors and underlying patterns influencing dam safety. Significant correlations were identified in dam safety monitoring, with variations in X9 demonstrating exceptionally strong associations with multiple monitoring parameters (*p* < 0.001). Similarly, X8 and X2 exhibited comparably robust correlations (*p* < 0.001), indicating their critical importance in dam safety assessments. Contrary to conventional analytical expectations that typically group X1, X2, and X3 within the same classification cluster, our cluster analysis revealed a distinct pattern where X2 demonstrated stronger affinity with X5 and X7. Principal Component Analysis (PCA) revealed that the first two principal components (PC1 and PC2) collectively accounted for over 70% of the total variance. The comprehensive safety assessment identified Section T4 as the highest-scoring segment (score: 0.8500), followed by T2 (0.8155), with T35 being the lowest (0.0175). These results not only provide a theoretical foundation for rapidly locating critical dam sections affecting structural safety but also clarify the correlations and relative impacts among diverse monitoring parameters, thereby advancing the mechanistic understanding of hydraulic structure behavior.

## Data Availability

The datasets generated during this study are available from the corresponding au-thor (Weixing Yang; email: 75604366@qq.com) upon reasonable request.
